# Authors' reply

**DOI:** 10.4103/0019-5413.40266

**Published:** 2008

**Authors:** Ashutosh P Mavalankar, Darshan Majmundar, Shubha Rani

**Affiliations:** *Dr. Jivraj Mehta Hospital, Ahmedabad - 380 007, India.*; 1 *B. V. Patel PERD Centre, Ahmedabad - 380 007, India.*

Sir

We would like to thank Dr. Maini L^1^ for showing interest in our paper.

1. We do not state that the 7.2% incidence of deep vein thrombosis (DVT) following lower limb surgery is insignificant. We have concluded that this incidence is much lower than what has been reported in the Western population.
2. The author has also cited some articles published from our country on the subject. Out of the six studies he has quoted by Shead, Nagi, Sharma, N. Rajgopalan Bhan, Maini with reported incidences 28%, 8%, 19.6%, 23.3%, 7.8%, 9.9% respectively, three studies show incidence of DVT very close to what we have reported.
3. We agree that age has been quoted as one of the important risk factors in the literature. However, some of the recent studies have not found it significant.^2^ In our study, only 13 patients out of total 125 patients included in the trial were below the age of 50 years. None of these patients developed postoperative DVT. So even if these 13 patients are excluded from the study, the incidence of DVT would be marginally higher i.e. 8% (nine patients out of 112 patients) which would be comparable to studies by Nagi, Bhan and Maini.
4. Out of 29 patients who underwent miscellaneous surgeries like fixation of acetabular fractures (1), Interlocking tibial nailing (5), interlocking femoral nailing (6), fixation of patellar fractures (3), fixation of ankle fractures (7), fixation of tibial plateau fractures (4), fixation of distal femoral fractures (3), only one patient who was operated for internal fixation of acetabular fracture developed postoperative DVT. If we exclude 28 of these 29 patients who did not develop postoperative DVT from the present study, the incidence of DVT would be 9.3% (nine patients out of 97 patients undergoing total hip or knee replacements or surgeries for fractures around the hip joint). This incidence is still much lower than what has been reported in the Western literature.
5. We also agree that isolated calf thrombi must not be ignored and they could perhaps be treated with antiplatelet agents in low-risk patients.^2^ There is some evidence in the literature to show that isolated calf thrombi are less likely to give rise to pulmonary embolism compared to proximal thrombi.
6. We would like to point out that during the year 2007, a few articles have been published which have cautioned against the regular use of Low Molecular Weight Heparin (LMWH) and warfarin in all the patients undergoing lower limb arthroplasty. Dorr *et al*.,^2^ reported 35 times higher prevalence of bleeding and hematomas in their study of 1179 consecutive total joint arthroplasties in 970 patients who had undergone primary and revision total hip and total knee replacement and managed with chemoprophylaxis (for high-risk patients) or for treatment. Several other studies have shown excellent efficacy of antiplatelet agents like Aspirin and Dipyridamole^2^–^5^ along with mechanical foot pump devices for prevention of postoperative DVT thereby avoiding bleeding complications associated with LMWH and warfarin.
7. To conclude we would like to stress that the purpose of our paper is not to undermine the importance and seriousness of postoperative DVT and its potential grave consequences. As stated in the paper, we do not make any definite recommendation as regards the prophylactic measures. However, we believe that orthopedic surgeons should refrain themselves from indiscriminate use of LMWH as the panacea for DVT due to its serious potential side-effects. It should be used in patients where clinical benefits clearly appear to outweigh the risks of bleeding complications. In the majority of low-risk patients, multimodal prophylaxis with mechanical devices and antiplatelet agents could substantially reduce the risk of this potentially fatal condition.
8. We also hope that large-scale studies involving many centers throughout our country would be conducted by an independent body without any financial interest to bring out the true incidence of this condition in the Indian population. This will not only help us identifying the risk factors for the development of this condition but also help us to lay down guidelines for prophylaxis.
